# Simultaneous sentinel lymph node computed tomography and locoregional chemotherapy for lymph node metastasis in rabbit using an iodine-docetaxel emulsion

**DOI:** 10.18632/oncotarget.15679

**Published:** 2017-02-24

**Authors:** Honsoul Kim, Eun-Ji Jang, Sang Kyum Kim, Woo Jin Hyung, Dong Kyu Choi, Soo-Jeong Lim, Joon Seok Lim

**Affiliations:** ^1^ Department of Radiology, Yonsei University College of Medicine, Seoul, Republic of Korea; ^2^ Research Institute of Radiological Science, Yonsei University College of Medicine, Seoul, Republic of Korea; ^3^ Department of Integrative Bioscience and Bioengineering, Sejong University, Seoul, Republic of Korea; ^4^ Department of Pathology, Yonsei University College of Medicine, Seoul, Republic of Korea; ^5^ Department of Surgery, Yonsei University College of Medicine, Seoul, Republic of Korea; ^6^ New Drug Development Center, Daegu-Gyeongbuk, Medical Innovation Foundation, Daegu, Republic of Korea

**Keywords:** sentinel lymph node, computed tomography (CT), iodine-docetaxel emulsion, lymph node metastasis, locoregional chemotherapy

## Abstract

**Purpose:**

A sentinel lymph node (SLN) tracer can gain multi-functionality by combining it with additional components. We developed a SLN tracer consisting of iodine and docetaxel and applied it as a theragnostic nanoparticle to simultaneously perform SLN computed tomography (CT) lymphography and locoregional chemotherapy of the draining lymphatic system.

**Results:**

Docetaxel could be loaded in iodine emulsions at a drug-to-surfactant weight ratio as high as that in the drug formulation Taxotere^®^. The particle size and drug concentration were stable during storage for up to 3 months in optimized nanoemulsions. Popliteal LN enhancement on CT was observed in all healthy rabbits (n=3) and VX2 tumor-implanted rabbits (n=6) 12 hours after injection. The rate of SLN metastasis was significantly lower in the treatment group (29.4%, 5/17) than in the non-treatment group (70.6%, 12/17) (P=0.038).

**Material and Methods:**

We prepared a nanoemulsion carrying both iodine and docetaxel in a single structure by optimizing the composition of surfactants surrounding the inner iodized oil core. CT was performed 12 hours after subcutaneous injection of the emulsion in healthy rabbits (n=3) and VX2 tumor-implanted rabbits (n=6) for SLN imaging. Next, we tested the effect of treatment by histopathologically assessing the popliteal LN metastasis rate in VX2 tumor-implanted rabbits 7 days after subcutaneous injection of the emulsion (treatment group, n=17) and comparing it with that of non-treatment group rabbits (n=17).

**Conclusions:**

We developed an iodine-docetaxel emulsion and demonstrated that it can be applied to simultaneously achieve CT SLN imaging and local chemotherapy against nodal metastasis.

## INTRODUCTION

Regional lymph nodes (LNs) are one of the most frequent sites of early carcinoma metastasis. The presence of regional LN metastasis not only reflects a more malignant nature of the tumor, but also is a potential source of subsequent distant metastasis [1]. It has been reported that improved local therapies can lead to better treatment outcomes in breast cancer, gastric cancer, rectal cancer, and malignancies arising in other organs [1–4]. However, as non-surgical or minimally invasive treatments are increasingly performed, the diagnosis and treatment of regional LN metastasis becomes extremely challenging because these procedures often omit LN dissection. Therefore, strategies to effectively diagnose and eradicate nodal metastatic tumor cells are extremely important, especially if a non-surgical and/or minimally invasive treatment option is considered.

The sentinel lymph node (SLN) is defined as the first LN that directly receives lymph flow from the primary tumor, and is the most likely site of early nodal metastasis. If the SLN is free from metastasis, the risk of metastasis to other LNs is considered low [5]. Therefore, SLN biopsy has been established as an effective method to determine whether surgical LN dissection is necessary, especially in breast cancer and malignant melanoma, and to a lesser extent in other kinds of tumors [6–8].

In practice, the basic strategy to identify the SLN is to perform peri-tumoral injection of lymphatic tracers and subsequently detect the LNs that uptake the tracers. Traditionally, radioactive colloids and vital dyes have been the most popular lymphatic tracers used. However, in spite of their usefulness, these tracers also have technical limitations, such as rapid wash away of vital dyes and poor resolution for radioactive colloids [9]. To enhance the feasibility and expand the applicability of SLN detection, several groups, including ours, have recently produced and tested various SLN tracers based on various imaging modalities, including but not limited to CT [9–11], MRI [12, 13], contrast-enhanced ultrasonography [14], and optical imaging [9, 15].

In our previous work, we synthesized an emulsion consisting of iodine and indocyanine green that served as a SLN tracer that enabled hybrid imaging by CT and optical imaging [9]. Such experience led us to hypothesize that multiple components with different roles can be packaged into a single SLN tracer to achieve multi-functionality. We speculated that the scope of SLN tracer use may be expanded beyond diagnosis to treatment of nodal metastasis if we produced a theragnostic emulsion by marinating a cytotoxic agent into the scaffold of the SLN tracer. With this approach, SLN imaging based on such a tracer would accomplish not only SLN detection, but also chemotherapy that selectively targets the draining lymphatic system, which might carry metastatic tumor cells.

The purpose of this study was to synthesize a theragnostic emulsion containing both iodine and docetaxel and apply it to a rabbit tumor model, to achieve simultaneous SLN detection by CT lymphography and locoregional chemotherapy against microscopic metastasis within the draining lymphatic system.

## RESULTS

### Physicochemical characterization of the iodine-docetaxel emulsion

The optimal combination of surfactants with a high and low hydrophilic-lipophilic balance value is critical for achieving a stable and fine emulsion [16]. To produce an oil-in-water type emulsion with nanoscale particles, Tween 80 and Span 85 were selected as the major hydrophilic surfactant and lipophilic cosurfactant, respectively. Tween 80 was also selected because it is the major surfactant used in Taxotere^®^, a commercially available docetaxel product.

We first prepared nanoemulsions of different surfactant compositions with a fixed total amount of surfactant (240 μmole) to investigate the optimal hydrophilic/hydrophobic surfactant ratio in terms of the loaded docetaxel concentration and particle size of the resultant emulsions. When nanoemulsions were prepared with increasing Tween 80 content (25 to 87 mol%), the loaded docetaxel content increased, but the mean particle size of the emulsions decreased in a Tween 80 content-dependent manner (Supplementary Table I). The polydispersity index (PI) of all emulsions was below 0.3, showing a homogenous distribution of emulsions regardless of surfactant composition. Emulsions with a >5 mg/ml loaded docetaxel concentration and <100 nm mean particle size could be obtained with 240 μmole total surfactant composed of an 87:13 Tween 80:Span 85 mixture; we adopted this composition for the synthesis of iodine-docetaxel emulsions for further experiments. In case of Tween 80:Span 85 micelles (without an oil core), the concentration of loaded docetaxel and the mean particle size were similar to those of Tween 80:Span 85 emulsions. These data indicate that, although docetaxel is somewhat soluble in Lipiodol (data not shown), the presence of Lipiodol did not further increase docetaxel loading.

Reducing the total surfactant amount may be beneficial for reducing surfactant-related side effects, such as hypersensitivity reactions after administration in cancer patients. We therefore investigated the effect of the total amount of surfactant mixture on the loaded docetaxel concentration, mean droplet size, and PI of the nanoemulsions. As shown in Supplementary Table I, reducing the total amount of surfactant from 240 to 60 μmole at a fixed Tween 80:Span 85 ratio decreased the loaded docetaxel concentration by 3.7-fold while increasing the particle size by 2.2-fold. These data indicate that the loaded docetaxel concentration was very dependent on the total surfactant amount, and 240 μmole of surfactant mixture per milliliter of emulsion dispersions was required to produce iodine-docetaxel emulsions with nanoscale size.

**Figure 1:**
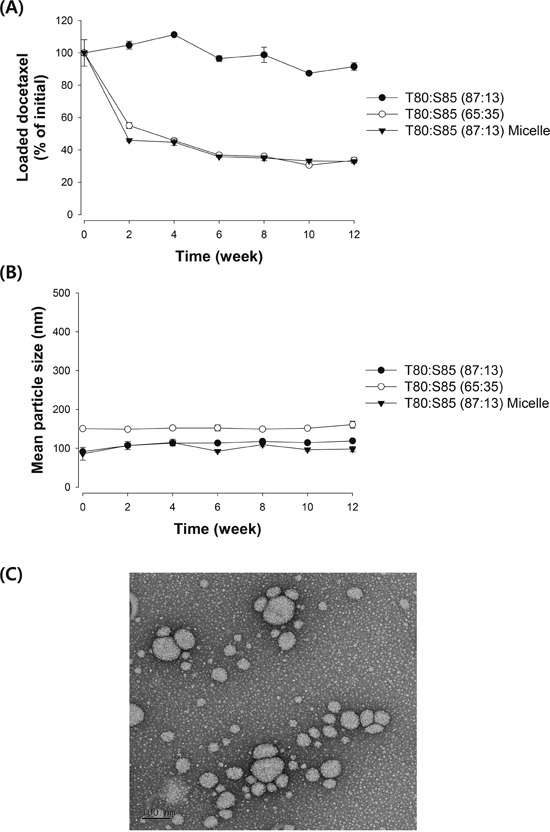
Emulsion stability assessed by changes in **(A)** loaded docetaxel concentration and **(B)** mean particle size of emulsions. The loaded docetaxel concentration of emulsions stored at 4°C was determined by HPLC analysis and change in droplet size was measured using a dynamic light scattering method. Data are presented as mean ± S.D. (n=3). **(C)** Representative TEM image of an iodine-docetaxel emulsion obtained with an 87:13 mixture of Tween 80:Span 85.

Because the use of nanoparticles as a carrier for taxane drugs, including docetaxel, is often hampered by the time-dependent release/precipitation of the drug [16], the stability of iodine-docetaxel emulsions was tested by measuring time-dependent changes in mean particle size and concentration of loaded docetaxel. Docetaxel loaded in emulsions containing 65% Tween 80 tended to precipitate in a time-dependent manner during storage at 4°C. Greater than 40% of loaded docetaxel was released/precipitated from micelles after 2 weeks of storage (Figure 1A). In contrast, nanoemulsions containing 87% Tween 80 showed greatly improved stability; no significant loss of docetaxel loaded in nanoemulsions was observed after storage for up to 8 weeks at 4°C. With regard to the mean particle size, no significant change in the particle size was found during storage up to 12 weeks, regardless of formulation (Figure 1B). Collectively, these results suggest that nanoemulsions with an inner iodized oil core stabilized by an 87:13 mixture of Tween 80 and Span 85 are a potential vehicle for stably carrying docetaxel.

The diameter and morphology of the emulsion obtained with the 87:13 mixture of Tween 80:Span 85 were also determined by TEM. As demonstrated by the representative TEM image shown in Figure 1C, the majority of the particles in this emulsion are spherical and less than 100 nm in size, in agreement with the results obtained by the dynamic light scattering method.

### CT lymphography for SLN imaging

In our previous study, we demonstrated that water-soluble emulsions containing iodine can serve as an effective CT lymphography tracer [9]. Our newly-synthesized iodine-docetaxel emulsion was similar to the previous versions in design and synthesis protocol, but differed in composition. Therefore, we first intended to verify whether the iodine-docetaxel emulsion would reliably serve as a CT lymphography agent, by testing in both healthy rabbits and microscopic LN metastasis rabbit models.

The LN metastasis model was generated by passage of tumors from the popliteal LN of VX2 tumor-implanted rabbits to additional heathy rabbits. Initially, the passage of VX2 tumors harvested from the popliteal LN was successful in no more than 50% of the rabbits, but the success rate gradually increased as we repeated passaging the tumors (data not shown). We believe that a tumor subpopulation with an enhanced tendency for nodal metastasis was selected for during passaging, causing the rate of popliteal LN metastasis to gradually increase. After several cycles of tumor passaging, we were able to establish a VX2 tumor line that showed enhanced nodal metastasis potential and usually achieved a popliteal LN metastasis rate of approximately 90% (data not shown) when incubated for 4 weeks after implantation. All subsequent experiments were performed using this VX2 tumor line.

**Figure 2:**
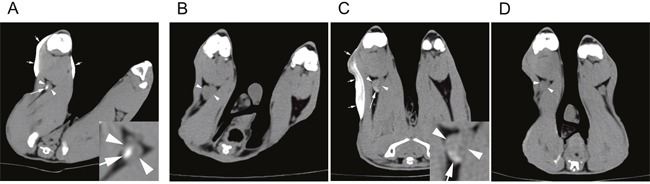
CT lymphography after subcutaneous injection of an iodine-docetaxel emulsion for sentinel lymph node (SLN) imaging in rabbit models Lower extremity CT of a **(A)** healthy control rabbit performed 12 hours after iodine-docetaxel emulsion injection. Multiphasic CT lymphography images of a VX2 tumor-implanted rabbit model obtained **(B)** before, **(C)** 12 hours after, and **(D)** 7 days after iodine-docetaxel emulsion injection. Focal intra-nodal contrast enhancement (arrow) within the popliteal LN (arrowheads) caused by the subcutaneously injected iodine-docetaxel emulsion (small arrows) was observed. Magnified images of the ipsilateral popliteal images showing focal uptake of iodine-docetaxel emulsion (Inset) are provided.

We performed CT lymphography in tumor-free healthy rabbits (n=3). CT images obtained 12 hours after subcutaneous injection of the iodine-docetaxel emulsion demonstrated focal contrast enhancement within the popliteal lymph node ipsilateral to the tumor (Figure 2A).

Next, we conducted SLN imaging in the rabbit microscopic LN metastasis model (n=6). Similar to the tumor-free healthy rabbits, CT lymphography was performed 12 hours after subcutaneous injection of the iodine-docetaxel emulsion, which also revealed focal intra-nodal enhancement by the uptake of emulsion (Figure 2B, 2C). A second session of CT lymphography was performed 7 days later and showed that the iodine-docetaxel emulsion washed away, as intranodal enhancement was no longer present (Figure 2D).

### SLN CT volume measurement

**Figure 3:**
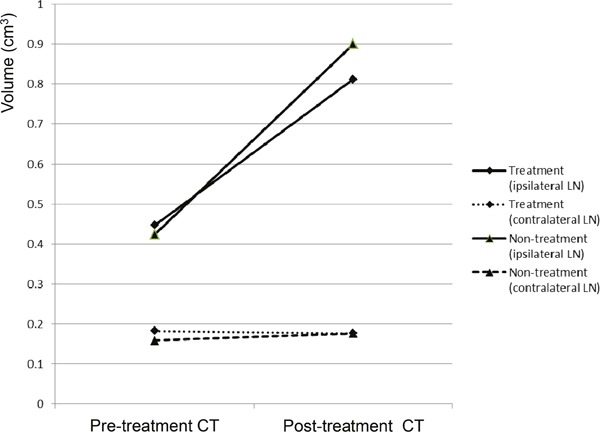
CT volumetry of the popliteal LNs of VX2-implanted rabbits performed before (pre-treatment CT) and 7 days after (post-treatment CT) injection The rabbits were divided into the treatment group (n=17), which received subcutaneous injection of the iodine-docetaxel emulsion, or the non-treatment (control) group (n=17).

The pre-treatment CT images obtained 10 days after VX2 tumor implantation were used for volumetry. The rabbits were alternatively allocated into the treatment group (n=17) or non-treatment (control) group (n=17) according to the size of the popliteal LN ipsilateral to the implanted tumor so that the two groups had matched LN size (mean ± standard deviation: treatment group, 0.424 ± 0.130 cm^3^ vs. non-treatment group, 0.447 ± 0.137 cm^3^; P=0.612). The size of the contralateral side popliteal LNs were also the same between the two groups upon allocation (mean ± standard deviation: treatment group, 0.159 ± 0.146 cm^3^ vs. non-treatment group, 0.182 ± 0.102 cm^3^; P=0.589) (Figure 3). Shortly after the pre-treatment CT was obtained, the treatment group rabbits received a peri-tumoral injection of the iodine-docetaxel emulsion.

Seven days after injection of the iodine-docetaxel emulsion in the treatment group (17 days after VX2 tumor implantation), a post-treatment CT was performed. Volumetry based on the post-treatment CT revealed that the sizes of the ipsilateral LNs in the treatment group compared to the non-treatment group were not significantly different (mean ± standard deviation: treatment group, 0.900 ± 0.283 cm^3^ vs. non-treatment group, 0.812 ± 0.193 cm^3^; P=0.296). Meanwhile, the sizes of the ipsilateral popliteal LNs significantly increased in both the treatment and non-treatment groups when the size measured on the post-treatment CT was compared with that of the pre-treatment CT within each group (paired t-test, P<0.001 in both groups) (Figure 3). However, the volume of the contralateral popliteal LN measured on the post-treatment CT did not differ between the two groups (mean ± standard deviation: treatment group, 0.176 ± 0.217 vs. non-treatment group, 0.176 ± 0.090; P=1.000), nor did it differ when comparing the pre-treatment CT and post-treatment CT of either group (paired t-test, within treatment group P=0.332; within non-treatment group P=0.332) (Figure 3).

### Treatment response measurement

**Figure 4:**
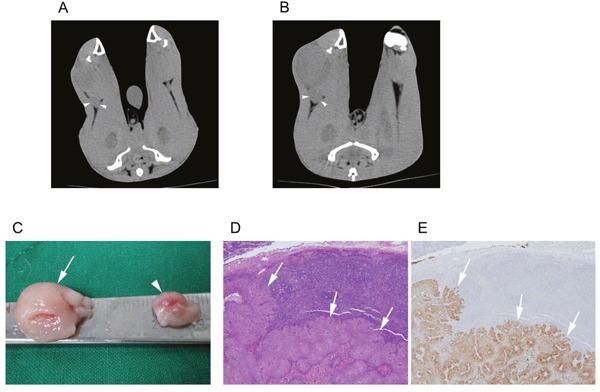
A non-treatment (control) group VX2-implanted rabbit Images obtained during **(A)** the pre-treatment CT session (10 days after tumor implantation) and **(B)** the post-treatment CT session (17 days after tumor implantation). The popliteal LN (arrowhead) ipsilateral to the implanted tumor increased in size from 0.4 to 0.9 cm^3^. **(C)** Gross image of the dissected popliteal LNs at the ipsilateral (arrow) and contralateral (arrowheads) side of the tumor. Microscopic images of **(D)** hematoxylin and eosin staining (40×) and **(E)** anti-cytokeratin (AE1+AE3 antibody) immunohistochemistry staining (40×) of the ipsilateral popliteal LN demonstrated tumor metastasis (arrows).

**Figure 5:**
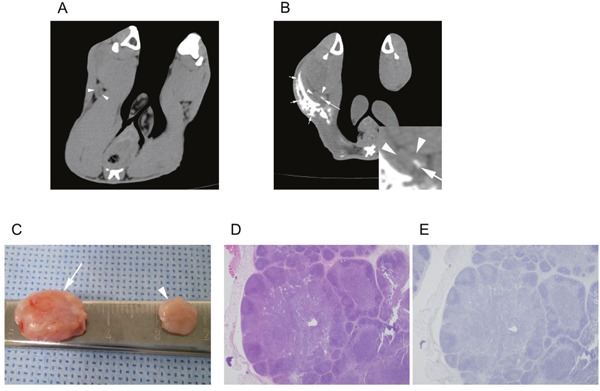
A treatment group VX2-implanted rabbit Images obtained during **(A)** the pre-treatment CT (10 days after tumor implantation) and **(B)** the post-treatment CT (17 days after tumor implantation, 7 days after iodine-docetaxel emulsion injection). The popliteal LN (arrowheads) ipsilateral to the implanted tumor increased in size from 0.3 to 0.6 cm^3^, and focal intra-nodal contrast enhancement (arrow) by uptake of the injected iodine-docetaxel emulsion (small arrows) was observed. Magnified image of the ipsilateral popliteal images showing focal uptake of iodine-docetaxel emulsion (Inset) is provided. **(C)** Gross image of the dissected popliteal LNs at the ipsilateral (arrow) and contralateral (arrowhead) side of the tumor. Microscopic images of **(D)** hematoxylin and eosin staining (40×) and **(E)** anti-cytokeratin (AE1+AE3 antibody) immunohistochemistry staining (40×) of the ipsilateral popliteal LN revealed no evidence of tumor metastasis.

**Figure 6:**
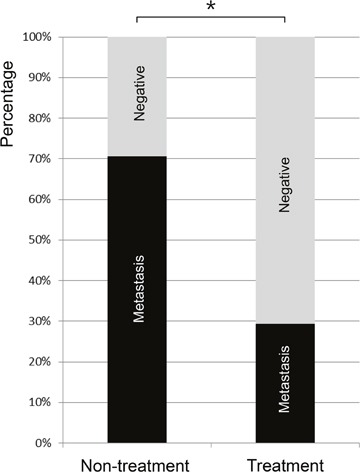
The rate of popliteal LN metastasis in VX2 tumor-implanted rabbits as detected by microscopic examination 17 days after tumor implantation (or 7 days after iodine-docetaxel emulsion injection in the treatment group) The rate of popliteal LN metastasis was 29.4% (n=5/17) in the treatment group versus 70.6% (n=12/17) in the non-treatment group, indicating that LN metastasis occurred less frequently in the treatment group (*P=0.038).

Both the non-treatment group (Figure 4) and treatment group rabbits (Figure 5) had an increased volume of the ipsilateral popliteal LNs compared with that of the contralateral popliteal LNs (Figure 3, 4C, 5C). Microscopic examination of the dissected ipsilateral popliteal LNs detected LN tumor metastasis more frequently in the non-treatment group than the treatment group (Figure 4D, 4E, 5D, 5E, 6). The ratio of positive LN metastasis was significantly lower (P=0.038) in the treatment group, as 70.6% (n=12/17) of the non-treatment group rabbits developed positive LN metastasis, compared with 29.4% (n=5/17) in the treatment group (Figure 6). The volumetry based on the post-treatment CT did not show a statistically significant difference according to the presence or absence of metastasis (mean ± standard deviation: LN positive for metastasis, 0.882 ± 0.260 cm^3^ vs. negative for metastasis, 0.829 ± 0.229 cm^3^; P=0.533).

## DISCUSSION

In the current study, we developed an iodine-docetaxel emulsion and demonstrated that it can be applied as a SLN lymphography tracer and a vehicle that selectively delivers a chemotherapy agent into the tumor draining lymphatic system. We showed that peri-tumoral injection of our iodine-docetaxel emulsion can significantly reduce the rate of LN metastasis in a LN micro-metastasis model (VX2 tumor-implanted rabbits).

In our previous study, we developed a water-soluble emulsion containing iodized oil and fluorescent dye and demonstrated that it can be used to perform SLN hybrid imaging with both CT and optical devices [9]. Based on such experience, we learned that a variety of emulsions can be produced to generate tracers with additional capabilities. We also previously showed that water-soluble emulsions with optimal particle size will accumulate within the draining LNs, instead of being rapidly washed away like the classical vital dye tracers used for SLN detection [9]. We hypothesized that if the water-soluble emulsion is mixed with a cytotoxic agent, then the tumor draining lymphatic system would become selectively exposed to the cytotoxic agent in high concentration as the tracer accumulates. Consequently, a fortified chemotherapy regimen specifically targeting the compartment of the draining lymphatic system could be established. To fulfill this scenario, we integrated the chemotherapy agent docetaxel into the scaffold of our previous version of emulsion designed as an SLN tracer. We believe that our approach can not only enable SLN detection, but also actively eradicate microscopic LN metastasis.

The capability to simultaneously perform SLN imaging and locoregional chemotherapy of the draining lymphatic system has several clinical implications and advantages. Most importantly, under usual clinical settings a surgical biopsy may not be feasible even if SLN imaging is practically possible. Nowadays, minimally invasive procedures, such as endoscopic resection or trans-anal operation, are increasingly performed for the treatment of early gastric cancer or early colorectal cancer, but the prediction and treatment of potential nodal metastasis remains a challenge because LN biopsy is not possible during these procedures [17, 18]. However, peri-tumoral injection of SLN tracers can be easily performed during these minimally invasive procedures along with a subsequent CT lymphography to perform SLN imaging. The treatment effect of our iodine-docetaxel emulsion can reduce the risk of potential microscopic LN metastasis, which we believe is a significant advantage because such minimally invasive endoscopic resection procedures stand on the assumption that tumor cells have not entered the draining lymphatic system, despite the fact that the LNs remain unexamined.

In early gastric cancer, the risk of LN metastasis has been reported to range between 1% to 3% if the tumor is confined to the mucosa, and 11% to 20% if the tumor invades the submucosa [18, 19]. Currently, endoscopic resection is considered as standard treatment in carefully selected patients who meet the absolute indication (differentiated type adenocarcinoma without ulcer and the depth of invasion is clinically diagnosed as T1a and the diameter is ≤2cm) because the risk of positive LN metastasis is low in these patients [20]. Meanwhile, ongoing debate exists on whether or not endoscopic resection can be safely performed in early gastric cancer beyond the absolute indication due to the risk of regional LN metastasis [21–23]. We believe that our iodine-docetaxel emulsion, which can be easily injected around the tumor during endoscopic resection, can be applied to control any undetected microscopic LN metastasis and therefore contribute to expand the application of such minimally invasive treatments. Moreover, this approach also can detect the SLN (or SLN basin) by CT, which can be used if a SLN biopsy is later decided to be necessary.

Our iodine-docetaxel emulsion can also improve safety in patients who decide not to undergo curative resection based on a negative SLN biopsy. Although SLN biopsy is an established method in several kinds of malignancies, the risk of a false negative report cannot be eliminated for several reasons. Firstly, during daily practice, a whole-mount examination of the retrieved SLN cannot be performed, and thus under-sampling is inevitable. Secondly, the current SLN detection methods have a risk of technical failure. Water-soluble dyes, including vital dyes, not only rapidly wash away and therefore allow only limited time for assessment, but may become obscured when covered by dense fat tissue. Radioactive colloid tracers have poor spatial resolution and are known to exhibit poor sensitivity when the LN is near the injection site because of high background scattering (shine-through effect) [24–26].

According to clinical reports, the majority of metastatic LNs discovered in colorectal and gastric cancer patients were not significantly enlarged [27–29], which is why the prediction of nodal metastasis is extremely challenging. The presence of an overt metastatic deposit (and consequently an unequivocally enlarged LN) would frequently occlude the lymphatic channels, causing SLN imaging based on tracer injection to be inaccurate. However, we believe that larger LNs would likely carry metastatic deposits with a tumor burden at a level that is unreasonable to attempt treatment based on a non-surgical method, including our iodine-docetaxel emulsion injection method. Therefore, we excluded rabbits with large popliteal LNs (>1 cm^3^) at pre-treatment CT.

In the current study, the size of the ipsilateral LNs measured on post-treatment CT increased during the 7 day incubation period in both the treatment group and non-treatment group (P<0.001 in both) when compared with the pre-treatment CT. However, in spite of the difference in the nodal metastasis rate between the treatment group and non-treatment group, the size of the LNs of the two groups was not significantly different (mean ± standard deviation: treatment group, 0.900 ± 0.283 cm^3^ vs. non-treatment group 0.812 ± 0.193 cm^3^; P=0.296). This implies that although treatment with the iodine-docetaxel emulsion might lower the rate of nodal metastasis, the treatment response is not reflected by a decrease in size of a particular LN, which would make it difficult to predict eradication of potential metastasis based on size alone. We believe that the increased size of non-metastatic LNs is probably associated with the state of reactive hyperplasia [29, 30].

It has been reported that docetaxel can reach much higher concentrations when packed in lipid emulsion form [31]. Docetaxel is a lipophilic agent, and the lipid emulsion can serve as a drug carrier that allows for the slow release of docetaxel [31]. A previous study showed that lipophilic drugs formulated in lipid emulsions can reach higher plasma concentrations than those in solution after intravenous injection [32]. Thus, by synthesizing our tracer in lipid emulsion form, we attempted to achieve similar pharmacokinetic benefits and speculated that the concentration of docetaxel within the draining LNs could be maximized.

Our formulation study, which was performed to optimize the composition of surfactants in order to maximize the loaded docetaxel concentration in the emulsion tracer, demonstrated that 240 μmole/ml of an 87:13 mixture of Tween 80:Span 85 enabled docetaxel loading at a 4% drug-to-surfactant weight ratio, a ratio comparable to that in Taxotere^®^, where 40 mg docetaxel is solubilized in an ethanol-surfactant mixture containing 1075 mg Tween 80 (3.6%). The mean size of optimized emulsions was less than 100 nm, the upper size limit for colloidal particles to enter the lymphatic capillaries quickly and accumulate in regional LNs after subcutaneous administration [33]. Moreover, they exhibited very high stability in terms of particle size and loaded docetaxel content during storage. Since docetaxel was retained as loaded in nanoemulsions but not in micelles obtained with the same Tween 80:Span 85 surfactant ratio, it seems likely that the presence of iodized oil contributed to inhibiting the docetaxel precipitation. We believe that the docetaxel was likely solubilized mainly in the iodized oil core together with surfactants; thus, the presence of iodized oil not only enables CT imaging but also stabilizes docetaxel-loaded emulsions.

We acknowledge that our study had a few limitations. Firstly, our study was based on a microscopic LN metastasis animal model in which the presence or absence of LN metastasis was unknown at the time of iodine-docetaxel emulsion injection. As a result, we were not able to measure the direct treatment effect on LN metastasis. To compensate for this limitation, we established a balanced control group with matched popliteal LN sizes, which served as a non-treatment (control) group for comparison. Secondly, to ensure the clarity of this proof-of-concept study, we performed experiments while using the popliteal LN as the SLN for the VX2 tumor implanted in the lower extremity. However, the lymphatic vessels of this area are very simple, and therefore we do not believe that our iodine-docetaxel emulsion has been sufficiently validated as a SLN tracer. We intend to pursue further validation in a more clinically relevant tumor model with more complex lymphatic structures.

In conclusion, we have developed an iodine-docetaxel emulsion and applied it to simultaneously accomplish SLN detection based on CT lymphography and eradication of potential LN metastasis by selectively delivering chemotherapy into the draining lymphatic system.

## MATERIALS AND METHODS

This study was approved by the Experimental Animal Ethical Committee of Yonsei University College of Medicine (approval number: 2013-0248).

### Preparation of nanoemulsions

Sorbitan trioleate (Span 85) and polyoxyethylenesorbitan monooleate (Tween 80) were purchased from Sigma-Aldrich Inc. (St. Louis, MO, USA). Docetaxel was purchased from LC laboratories (Woburn, MA, USA). Lipiodol, an FDA-approved iodized oil, was purchased from Guerbet (Aulnay-Sous-Bois, France). All other materials were of reagent grade and used without further purification.

Oil-in-water emulsions were prepared using a homogenization method as previously described [34]. Briefly, 7 mg of docetaxel and 300 μl of Lipiodol were dissolved in ethanol together with varying amounts of surfactant mixture (Tween 80 and Span 85). Ethanol was removed under reduced pressure using a rotary evaporator until a pre-emulsion concentrate was obtained. We then added 0.7 ml of saline dropwise to the pre-emulsion concentrate while vortexing, followed by sonication in a bath sonicator at 37°C for 30 min. The obtained emulsions were placed in an ice bath, then homogenized at 11,000 rpm using a homogenizer (IKA-Wereke, Stufen, Germany) in four intervals (durations: 10, 5, 5, and 5 min), to obtain a homogeneous nanoemulsion. Unloaded docetaxel was removed from the emulsion dispersion by filtering through an 0.8-μm syringe membrane filter. The filtered emulsions were stored at 4°C until use.

### Physicochemical characterization of emulsions

The mean particle size and PI of the nanoemulsions were measured by dynamic light scattering using a fiber-optic particle analyzer (FPAR-1000, Otsuka Electronics, Japan). Prior to measurement, the emulsion dispersions were appropriately diluted with filtered saline. The PI was obtained as a measure of the uniformity of the particle size distribution in colloidal dispersions [35].

The concentration of docetaxel loaded in the emulsions was determined by reversed-phase HPLC analysis [35]. In brief, 50 μl of each filtered emulsion was rapidly frozen, dried in a freeze dryer (FDU-1200, EYELA, Japan), then redissolved in methanol. Twenty microliters of sample were injected into the Nanospace SI-2HPLC system (Shiseido, Japan) equipped with a mobile phase delivery pump (SP 3201) and UV-vis detector (SP 3002). A mixture of acetonitrile and distilled water (65:35, v/v) was used as a mobile phase at a flow rate of 1 ml/min. A reversed-phase Capcell pak C18 column (UG120, Shiseido, Japan) was used at 35°C. The detector wavelength was set at 230 nm.

The diameter and morphology of nanoemulsions were also determined by transmission electron microscopy (TEM; Tecnai G2 Spirit, FEI) at an accelerator voltage of 120 kV. For observation, 5 μl of emulsion were applied to carbon-coated grids that had been glow discharged for 3 min in air and immediately (within ∼5 sec) negatively stained using 1% uranyl acetate.

The stability of nanoemulsions was evaluated by monitoring the changes in mean particle size and the docetaxel content remaining loaded in emulsions during storage at 4°C.

### Animal model of popliteal LN metastasis

Male New Zealand white rabbits weighing from 3 to 3.5 kg each were used. Anesthesia was induced by intramuscular injection of 5 mg of a 1:1 combination mixture of zolazepam (Zoletil; Vibrac Laboratories, Carros, France) and xylazine hydrochloride (Rompun; Bayer, Seoul, Korea) per kilogram body weight.

VX2 tumors exhibiting enhanced tendency to metastasize to the LN were selected through multiple rounds of passaging. Rabbits implanted with a VX2 tumor in the lower extremity muscle were incubated for 4 to 5 weeks to allow popliteal LN metastasis to occur. Under anesthesia, the popliteal LN ipsilateral to the implanted tumor (where it was assumed, but not confirmed, that nodal metastasis had developed) was harvested and minced into approximately 1-mm^3^ pieces. This fresh tissue suspension was implanted into another rabbit within the muscles at the outer side of the distal portion of right tibia at a total volume of 0.1 ml. The rabbit was incubated for 4 to 5 weeks until tumor passage to the next rabbit was performed.

### SLN imaging based on CT lymphography

First, we intended to test whether our iodine-docetaxel emulsion could serve as a SLN tracer. Under anesthesia, we subcutaneously injected the iodine-docetaxel emulsion at the right lower extremity in healthy control rabbits (n=3) and VX2 tumor-implanted rabbits (n=6, incubated for 10 days). Each rabbit received three 0.7-ml injections for 2.1 ml in total, either within a narrow area (healthy rabbits) or at a peri-tumoral location (tumor-implanted rabbits). CT images were obtained before and 12 hours after subcutaneous injection of the iodine-docetaxel emulsion. The VX2 tumor-implanted rabbits underwent a follow-up CT scan 7 days after this procedure.

CT lymphography was performed with a commercial 64-channel multidetector scanner (Discovery CT750 HD; GE Healthcare, Milwaukee, Wis) with the following parameters: 120 kV, 250 mA, 0.625-mm beam collimation, and 1.25-mm axial reconstruction thickness. The field of view was adjusted to include the entire abdomen and lower extremities.

### Treatment response measurement

Thirty-six New Zealand white rabbits implanted with a VX2 tumor in the muscle of right side lower limb and incubated for 10 days served as a model of microscopic LN metastasis. A CT scan was performed twice, just before peri-tumoral injection of the iodine-docetaxel emulsion (pre-treatment CT) and 7 days after injection (post-treatment CT). Volumetry of popliteal LNs was also conducted twice in each rabbit based on the pre-treatment CT and post-treatment CT.

For volume measurement of the popliteal LNs, the CT axial images were archived in Digital Imaging and Communications in Medicine (DICOM) format and stored on a General Electric Advantage v4 work station (GE Healthcare, Waukesha, WI). The region of interest (ROI) was drawn along the margin of the popliteal LN on each slice, and total mass was calculated by adding ROI areas in consecutive images. The volumes of popliteal LNs were measured on both the ipsilateral and contralateral side to the implanted tumor.

In humans, it is known that metastatic LNs tend to increase in size [36, 37], and we speculated that large LNs may represent LNs carrying gross metastasis deposits rather than microscopic metastases. As we tried to simulate a microscopic nodal metastasis model, we intended to exclude rabbits with LNs carrying overt metastasis at the time of SLN assessment. Therefore, we defined 1 cm^3^ as a volume threshold and excluded rabbits (n=2) in which the volume of their ipsilateral popliteal LNs exceeded 1 cm^3^ on pre-treatment CT. The remaining rabbits (n=34) were alternatively allocated into the treatment group (n=17) or non-treatment (control) group (n=17) according to the volume of the ipsilateral popliteal LN measured on pre-treatment CT. Shortly after the pre-treatment CT scan was obtained and volumetry was performed, the treatment group rabbits received peri-tumoral subcutaneous injections (three 0.7 ml injections, 2.1 ml in total) around the tumor nodule. Seven days later (17 days after the tumor was implanted), a post-treatment CT scan was conducted. Afterwards, all rabbits were euthanized with saturated potassium chloride and the bilateral popliteal LNs were sampled.

### Analysis of the dissected popliteal LNs

The sampled popliteal LNs were fixed in 10% buffered formalin overnight, then embedded in paraffin and sectioned at 5-mm intervals. We performed hematoxylin and eosin staining and immunohistochemical staining using anti-cytokeratin monoclonal antibodies (anti-cytokeratin AE1+AE3 antibody, Abcam Inc., Cambridge, MA). The slides were reviewed by a pathologist (K.S.K.) with 5 years of experience in experimental animal histopathology to determine the presence or absence of metastatic tumor cells.

### Statistical analysis

Statistical analysis was performed using standard software (SPSS v20.0 for Windows, SPSS Inc., Chicago, IL, USA). Student's t-test was used to compare the volume of popliteal LNs between the treatment group and non-treatment group. Paired t-test analysis was applied to compare the volumes of a popliteal LN measured on the pre-treatment and post-treatment CT scans. Fisher's exact test was performed to compare the rate of tumor LN metastasis between the treatment group and non-treatment group. Statistical significance was defined as P<0.05.

## SUPPLEMENTARY TABLE



## References

[1] Kawada K, Taketo MM (2011). Significance and mechanism of lymph node metastasis in cancer progression. Cancer Res.

[2] Clarke M, Collins R, Darby S, Davies C, Elphinstone P, Evans V, Godwin J, Gray R, Hicks C, James S, MacKinnon E, McGale P, McHugh T (2005). Effects of radiotherapy and of differences in the extent of surgery for early breast cancer on local recurrence and 15-year survival: an overview of the randomised trials. Lancet.

[3] Wu CW, Hsiung CA, Lo SS, Hsieh MC, Chen JH, Li AF, Lui WY, Whang-Peng J (2006). Nodal dissection for patients with gastric cancer: a randomised controlled trial. Lancet Oncol.

[4] Kobayashi H, Mochizuki H, Kato T, Mori T, Kameoka S, Shirouzu K, Sugihara K (2009). Outcomes of surgery alone for lower rectal cancer with and without pelvic sidewall dissection. Dis Colon Rectum.

[5] Morton DL, Wen DR, Wong JH, Economou JS, Cagle LA, Storm FK, Foshag LJ, Cochran AJ (1992). Technical details of intraoperative lymphatic mapping for early stage melanoma. Arch Surg.

[6] Morton DL, Thompson JF, Cochran AJ, Mozzillo N, Elashoff R, Essner R, Nieweg OE, Roses DF, Hoekstra HJ, Karakousis CP, Reintgen DS, Coventry BJ, Glass EC (2006). Sentinel-node biopsy or nodal observation in melanoma. N Engl J Med.

[7] Park DJ, Lee HJ, Lee HS, Kim WH, Kim HH, Lee KU, Choe KJ, Yang HK (2006). Sentinel node biopsy for cT1 and cT2a gastric cancer. Eur J Surg Oncol.

[8] Linehan DC, Hill AD, Akhurst T, Yeung H, Yeh SD, Tran KN, Borgen PI, Cody HS (1999). Intradermal radiocolloid and intraparenchymal blue dye injection optimize sentinel node identification in breast cancer patients. Ann Surg Oncol.

[9] Kim H, Lee SK, Kim YM, Lee EH, Lim SJ, Kim SH, Yang J, Lim JS, Hyung WJ (2015). Fluorescent iodized emulsion for pre- and intraoperative sentinel lymph node imaging: validation in a preclinical model. Radiology.

[10] Lee YJ, Kim YH, Lee KH, Park JH, Lee HS, Jung SC, Joo SM (2014). Sentinel node mapping of VX2 carcinoma in rabbit thigh with CT lymphography using ethiodized oil. Korean J Radiol.

[11] Kim YH, Lee YJ, Park JH, Lee KH, Lee HS, Park YS, Park do J, Kim HH (2013). Early gastric cancer: feasibility of CT lymphography with ethiodized oil for sentinel node mapping. Radiology.

[12] Rubio IT, Diaz-Botero S, Esgueva A, Rodriguez R, Cortadellas T, Cordoba O, Espinosa-Bravo M (2015). The superparamagnetic iron oxide is equivalent to the Tc99 radiotracer method for identifying the sentinel lymph node in breast cancer. Eur J Surg Oncol.

[13] Thill M, Kurylcio A, Welter R, van Haasteren V, Grosse B, Berclaz G, Polkowski W, Hauser N (2014). The Central-European SentiMag study: sentinel lymph node biopsy with superparamagnetic iron oxide (SPIO) vs. radioisotope. Breast.

[14] Xie F, Zhang D, Cheng L, Yu L, Yang L, Tong F, Liu H, Wang S, Wang S (2015). Intradermal microbubbles and contrast-enhanced ultrasound (CEUS) is a feasible approach for sentinel lymph node identification in early-stage breast cancer. World J Surg Oncol.

[15] Schaafsma BE, Verbeek FP, Elzevier HW, Tummers QR, van der Vorst JR, Frangioni JV, van de Velde CJ, Pelger RC, Vahrmeijer AL (2014). Optimization of sentinel lymph node mapping in bladder cancer using near-infrared fluorescence imaging. J Surg Oncol.

[16] Immordino ML, Brusa P, Arpicco S, Stella B, Dosio F, Cattel L (2003). Preparation, characterization, cytotoxicity and pharmacokinetics of liposomes containing docetaxel. J Control Release.

[17] Althumairi AA, Gearhart SL (2015). Local excision for early rectal cancer: transanal endoscopic microsurgery and beyond. J Gastrointest Oncol.

[18] Gotoda T, Sasako M, Ono H, Katai H, Sano T, Shimoda T (2001). Evaluation of the necessity for gastrectomy with lymph node dissection for patients with submucosal invasive gastric cancer. Br J Surg.

[19] Nakajima T (2002). Gastric cancer treatment guidelines in Japan. Gastric Cancer.

[20] Japanese Gastric Cancer A (2011). Japanese gastric cancer treatment guidelines 2010 (ver 3). Gastric Cancer.

[21] Gotoda T, Yanagisawa A, Sasako M, Ono H, Nakanishi Y, Shimoda T, Kato Y (2000). Incidence of lymph node metastasis from early gastric cancer: estimation with a large number of cases at two large centers. Gastric Cancer.

[22] Ahn JY, Jung HY (2013). Long-term outcome of extended endoscopic submucosal dissection for early gastric cancer with differentiated histology. Clin Endosc.

[23] Shim CN, Lee SK (2014). Endoscopic submucosal dissection for undifferentiated-type early gastric cancer: do we have enough data to support this?. World J Gastroenterol.

[24] Kitagawa Y, Fujii H, Kumai K, Kubota T, Otani Y, Saikawa Y, Yoshida M, Kubo A, Kitajima M (2005). Recent advances in sentinel node navigation for gastric cancer: a paradigm shift of surgical management. J Surg Oncol.

[25] Kitagawa Y, Fujii H, Mukai M, Kubota T, Otani Y, Kitajima M (2002). Radio-guided sentinel node detection for gastric cancer. Br J Surg.

[26] Mariani G, Moresco L, Viale G, Villa G, Bagnasco M, Canavese G, Buscombe J, Strauss HW, Paganelli G (2001). Radioguided sentinel lymph node biopsy in breast cancer surgery. J Nucl Med.

[27] Brown G, Richards CJ, Bourne MW, Newcombe RG, Radcliffe AG, Dallimore NS, Williams GT (2003). Morphologic predictors of lymph node status in rectal cancer with use of high-spatial-resolution MR imaging with histopathologic comparison. Radiology.

[28] Herrera-Ornelas L, Justiniano J, Castillo N, Petrelli NJ, Stulc JP, Mittelman A (1987). Metastases in small lymph nodes from colon cancer. Arch Surg.

[29] Tatsumi Y, Tanigawa N, Nishimura H, Nomura E, Mabuchi H, Matsuki M, Narabayashi I (2006). Preoperative diagnosis of lymph node metastases in gastric cancer by magnetic resonance imaging with ferumoxtran-10. Gastric Cancer.

[30] Deserno WM, Harisinghani MG, Taupitz M, Jager GJ, Witjes JA, Mulders PF, Hulsbergen van de Kaa CA, Kaufmann D, Barentsz JO (2004). Urinary bladder cancer: preoperative nodal staging with ferumoxtran-10-enhanced MR imaging. Radiology.

[31] Zhao M, Su M, Lin X, Luo Y, He H, Cai C, Tang X (2010). Evaluation of docetaxel-loaded intravenous lipid emulsion: pharmacokinetics, tissue distribution, antitumor activity, safety and toxicity. Pharm Res.

[32] Patlolla RR, Vobalaboina V (2005). Pharmacokinetics and tissue distribution of etoposide delivered in parenteral emulsion. J Pharm Sci.

[33] Oussoren C, Storm G (2001). Liposomes to target the lymphatics by subcutaneous administration. Adv Drug Deliv Rev.

[34] Lee EH, Hong SS, Kim SH, Lee MK, Lim JS, Lim SJ (2014). Computed tomography-guided screening of surfactant effect on blood circulation time of emulsions: application to the design of an emulsion formulation for paclitaxel. Pharm Res.

[35] Hong SS, Kim SH, Lim SJ (2015). Effects of triglycerides on the hydrophobic drug loading capacity of saturated phosphatidylcholine-based liposomes. Int J Pharm.

[36] Kim JH, Beets GL, Kim MJ, Kessels AG, Beets-Tan RG (2004). High-resolution MR imaging for nodal staging in rectal cancer: are there any criteria in addition to the size?. Eur J Radiol.

[37] Choi J, Oh SN, Yeo DM, Kang WK, Jung CK, Kim SW, Park MY (2015). Computed tomography and magnetic resonance imaging evaluation of lymph node metastasis in early colorectal cancer. World J Gastroenterol.

