# Supplementary material for: Simultaneous sentinel lymph node computed tomography and locoregional chemotherapy for lymph node metastasis in rabbit using an iodine-docetaxel emulsion
Source: Oncotarget. 2017 Feb 24;8(16):27177–88. doi: 10.18632/oncotarget.15679 (PMC5432327; doi:10.18632/oncotarget.15679)
Supplement: Supplementary file 1 [file oncotarget-08-27177-s001.pdf]

Simultaneous sentinel lymph node computed tomography and locoregional chemotherapy for lymph node metastasis in rabbit using an iodine-docetaxel emulsion

SUPPLEMENTARY TABLE

Supplementary Table 1: Effect of surfactant composition on loaded docetaxel (DTX) concentration, mean particle size, and polydispersity index of iodine-docetaxel emulsions

Surfactant composition (mol%) (total µmole/ml)	Loaded DTX (mg/ml)	Mean droplet size (nm)	Polydispersity index
T80:S85 (87:13) (60 µmole)	1.63 ± 0.11	182 ± 16	0.251 ± 0.008
T80:S85 (87:13) (120 µmole)	5.34 ± 0.31	158 ± 3	0.238 ± 0.005
T80:S85 (87:13) (240 µmole)	5.82 ± 0.10	82 ± 3	0.234 ± 0.019
T80:S85 (65:35) (240 µmole)	5.77 ± 0.09	151 ± 4	0.202 ± 0.018
T80:S85 (45:55) (240 µmole)	2.80 ± 0.03	163 ± 4	0.224 ± 0.009
T80:S85 (25:75) (240 µmole)	1.18 ± 0.00	195 ± 30	0.209 ± 0.014
T80:S85 (87:13) Micelle (240 µmole)	5.14 ± 0.42	86 ± 16	0.329 ± 0.047

Data are presented as mean ± standard deviation (n=3). T80, Tween 80; S85, Span 85.
